# Editorial: Cellular and systemic interplay of metabolism and inflammation in the pathogenesis of lung diseases

**DOI:** 10.3389/fimmu.2023.1352304

**Published:** 2024-01-17

**Authors:** Adan Chari Jirmo, Melanie Albrecht, Slaven Crnkovic, Miguel Angel Alejandre Alcazar

**Affiliations:** ^1^ Department of Pediatric Pneumology, Allergology and Neonatology, Hannover Medical School, Hannover, Germany; ^2^ German Center for Lung Research (DZL), Hannover, Germany; ^3^ Paul-Ehrlich Institute (PEI), Langen, Germany; ^4^ Ludwig Boltzmann Institute (LBI) for Lung and Vascular Research, Otto Loewi Research Center Medical University of Graz, Graz, Austria; ^5^ Department of Pediatric and Adolescent Medicine, Faculty of Medicine and University Hospital Cologne, University of Cologne, Cologne, Germany; ^6^ Cologne Excellence Cluster on Cellular Stress Responses in Aging-Associated Diseases (CECAD) and Center for Molecular Medicine Cologne (CMMC), Faculty of Medicine and University Hospital Cologne, University of Cologne, Cologne, Germany; ^7^ German Center for Lung Research (DZL), Institute for Lung Health (ILH), University of Giessen and Marburg Lung Center (UGMLC) and Cardiopulmonary Institute (CPI), Marburg, Germany

**Keywords:** metabolism, metaflammation, inflammation, immuno-metabolism, lung pathologies

Research in the last couple of years has generated ample evidence showing that cellular alterations of the metabolic machinery could be the underlying factor in multiple human diseases. In this Research Topic, we have gathered scientific articles to underpin the interplay between perturbed metabolism/triggering of inflammation that contributes to pulmonary pathologies.

In this Research Topic, Wang et al., through a literature review, clearly illustrate the impact of various metabolic processes such as glycolysis, OXPHOS, FAO, and glutamine metabolism in modulating phenotypic and functional changes in macrophages and how these changes affect acute lung injury or acute respiratory distress syndrome. Moreover, through this review, Wang et al. reveal that metabolic reprogramming of macrophages is accompanied by dramatic shifts in cell metabolism and that functional state-associated unique metabolic signatures can be identified in various macrophage populations. Tang et al. have gone further in an original study to show that alveolar macrophages (AM) sensing of amino acid phenylalanine promotes pyroptosis, which causes the release of inflammatory mediators and, thereby, exacerbates lung inflammation and acute respiratory distress syndrome (ARDS) lethality in a murine model. This study is especially interesting since it not only elucidates the entire process of how phenylalanine initiates pyroptosis in AMs and the resulting inflammation but also elucidates the subsequent effects of the inhibition of this process on ARDS. The article clearly shows the significance of small-molecule metabolites in pulmonary inflammation and how they may be useful not only as biomarkers but as potential therapeutic targets. In their work, Li et al. expand this Research Topic further by showing how sensitivity to ferroptosis influences the inflammatory and lung repair capabilities of macrophages. They demonstrate that in chronic obstructive pulmonary disease (COPD), lipid peroxidation favors the differentiation of AM toward ferroptosis-sensitive M2-like macrophages but not ferroptotic-resistant M1-like macrophages. The Ferroptotic M2-like AMs lose their anti-inflammatory and repair functions but continue invoking inflammatory responses in COPD. Due to persistent lipid peroxidation in a COPD lung, this polarization toward M2-like AMs is speculated to be the cause of consistent inflammation and tissue damage. Importantly, the study shows that this process is therapeutically targetable since the ferroptotic phenotype can be ameliorated with anti-ferroptotic compounds, iron chelators, and heme oxygenase (HO-1) inhibitors and thereby alleviate lung inflammation, destruction, and remodeling of COPD.

More evidence of the impact of metabolic distortion on cellular homeostasis and how it influences lung pathology was provided by Bauer et al. They report that in severe cases of coronavirus disease-2019 (COVID-19), hypoxia-sensitized toll-like receptor 4 (TLR4) signaling activates SARS-CoV-2 spike protein in monocytes, ultimately leading to systemic inflammation in severe cases of COVID-19 as a result of enhanced chemokine ISG expression in monocytes upon infection with SARS-CoV-2. The study by Bauer et al. shows the connection between hypoxia-evoked disturbances in cholesterol metabolism and altered interferon (IFN) responses in monocytes and how this concomitantly affects inflammatory responses in the lung. Furthermore, the study provides an explanation regarding the possible mechanism of systemic inflammation which has been observed in severe cases of COVID-19 infections. In addition, Hasankhani et al. show in their review that metabolic perturbances are an underlying factor in the induction of the SARS-CoV-2-associated cytokine storm which is the main COVID-associated immunopathology that is related to disease severity and mortality.

Finally, Lim and Templeton in their mini-review highlight the important immunomodulatory function of hormones on the example of Adipokines exemplified through Adiponectin (APN). They speculate possible roles and mechanisms of Adiponectin (APN) pathway-induced protection in lung diseases, including fungal, bacterial, and viral infection, which could result in novel therapies that protect against infection, excessive inflammation, and other lung pathologies. This closes the potential circle and provides “food for thought” on how potentially feeding behavior and accompanying disturbances in hormones that regulate metabolism might influence or be exploited in the management of lung inflammatory conditions. Thus, with this Research Topic on *Cellular and Systemic Interplay of Metabolism and Inflammation in the Pathogenesis of Lung Diseases*, we provide an overview though not exhaustive of how metaflammation influences pulmonary immunity and pathologies and the need for intensification of research in this area.

## Author contributions

AJ: Conceptualization, Writing – original draft, Writing – review & editing. MA: Conceptualization, Writing – original draft, Writing – review & editing. SC: Conceptualization, Writing – original draft, Writing – review & editing. MA: Conceptualization, Writing – original draft, Writing – review & editing.

